# Photophobia and Blepharospasm Relieved by Chloroform

**Published:** 1857-11

**Authors:** 


*Photophobia and Blepharospasm relieved by Chloroform.*

Dr. Mackenzie, of Glasgow, communicated to the Royal Med. and Chir. Soc. a case of intense and long-continued (sixteen months) photophobia and blepharospasm relieved by the inhalation of chloroform administered seven times. Mr. Arnott stated that he had administered this remedy in a case of strumous ophthalmia, with intolerance of light, with not only immediate, but permanent relief.

